# 3-Acetyl-1-(2,4-dimethyl­phen­yl)thio­urea

**DOI:** 10.1107/S1600536812032382

**Published:** 2012-07-18

**Authors:** B. Thimme Gowda, Sabine Foro, Sharatha Kumar

**Affiliations:** a Department of Chemistry, Mangalore University, Mangalagangotri 574 199, Mangalore, India; b Institute of Materials Science, Darmstadt University of Technology, Petersenstrasse 23, D-64287 Darmstadt, Germany

## Abstract

In the crystal structure of the title compound, C_11_H_14_N_2_OS, the two N—H bonds are *anti* to each other. There is an intramolecular N—H⋯O hydrogen bond generating an *S*(6) ring motif.In the crystal, mol­ecules are linked *via* N—H⋯S hydrogen bonds with an *R*_2_^2^(8) motif and N—H⋯O hydrogen bonds with an *R*_2_^2^(12) motif into chains running along [1-10].

## Related literature

For studies on the effects of substituents on the structures and other aspects of *N*-(ar­yl)-amides, see: Gowda *et al.* (2001 ▶); Kumar *et al.* (2012 ▶); Shahwar *et al.* (2012 ▶), of *N*-(ar­yl)-methane­sulfonamides, see: Gowda *et al.* (2007 ▶) and of *N*-chloro­aryl­sulfonamides, see: Gowda & Ramachandra (1989 ▶); Shetty & Gowda (2004 ▶).
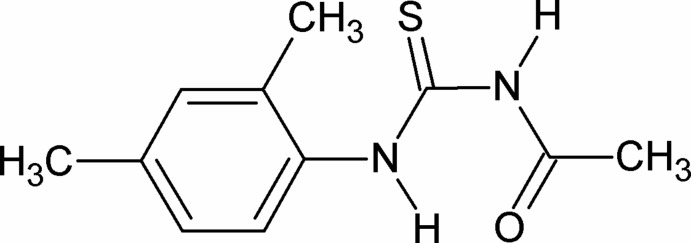


## Experimental

### 

#### Crystal data

C_11_H_14_N_2_OS
*M* *_r_* = 222.30
Triclinic, 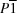

*a* = 5.0510 (7) Å
*b* = 9.973 (1) Å
*c* = 12.503 (2) Å
α = 69.15 (1)°
β = 89.43 (1)°
γ = 84.07 (1)°
*V* = 585.18 (14) Å^3^
*Z* = 2
Mo *K*α radiation
μ = 0.25 mm^−1^
*T* = 293 K
0.44 × 0.44 × 0.20 mm

#### Data collection

Oxford Diffraction Xcalibur diffractometer with a Sapphire CCD detector
Absorption correction: multi-scan (*CrysAlis RED*; Oxford Diffraction, 2009 ▶) *T*_min_ = 0.897, *T*_max_ = 0.951
3701 measured reflections
2393 independent reflections
2094 reflections with *I* > 2σ(*I*)
*R*_int_ = 0.008

#### Refinement

*R*[*F* ^2^ > 2σ(*F* ^2^)] = 0.037
*wR*(*F* ^2^) = 0.097
*S* = 1.07
2393 reflections
145 parameters
2 restraints
H atoms treated by a mixture of independent and constrained refinement
Δρ_max_ = 0.20 e Å^−3^
Δρ_min_ = −0.19 e Å^−3^

### 

Data collection: *CrysAlis CCD* (Oxford Diffraction, 2009 ▶); cell refinement: *CrysAlis CCD*; data reduction: *CrysAlis RED* (Oxford Diffraction, 2009 ▶); program(s) used to solve structure: *SHELXS97* (Sheldrick, 2008 ▶); program(s) used to refine structure: *SHELXL97* (Sheldrick, 2008 ▶); molecular graphics: *PLATON* (Spek, 2009 ▶); software used to prepare material for publication: *SHELXL97*.

## Supplementary Material

Crystal structure: contains datablock(s) I, global. DOI: 10.1107/S1600536812032382/bt5977sup1.cif

Structure factors: contains datablock(s) I. DOI: 10.1107/S1600536812032382/bt5977Isup2.hkl

Supplementary material file. DOI: 10.1107/S1600536812032382/bt5977Isup3.cml

Additional supplementary materials:  crystallographic information; 3D view; checkCIF report

## Figures and Tables

**Table 1:** Hydrogen-bond geometry (Å, °)

*D*—H⋯*A*	*D*—H	H⋯*A*	*D*⋯*A*	*D*—H⋯*A*
N1—H1*N*⋯O1	0.86 (1)	1.99 (2)	2.6673 (18)	135 (2)
N1—H1*N*⋯O1^i^	0.86 (2)	2.44 (2)	3.121 (2)	137 (2)
N2—H2*N*⋯S1^ii^	0.84 (1)	2.55 (2)	3.3711 (14)	168 (2)

Symmetry codes: (i) 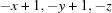; (ii) 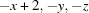.

**Fig. 1.**
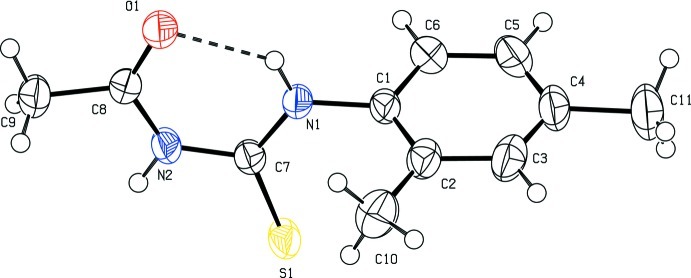
Molecular structure of the title compound, showing the atom labelling scheme and with displacement ellipsoids drawn at the 50% probability level.

**Fig. 2.**
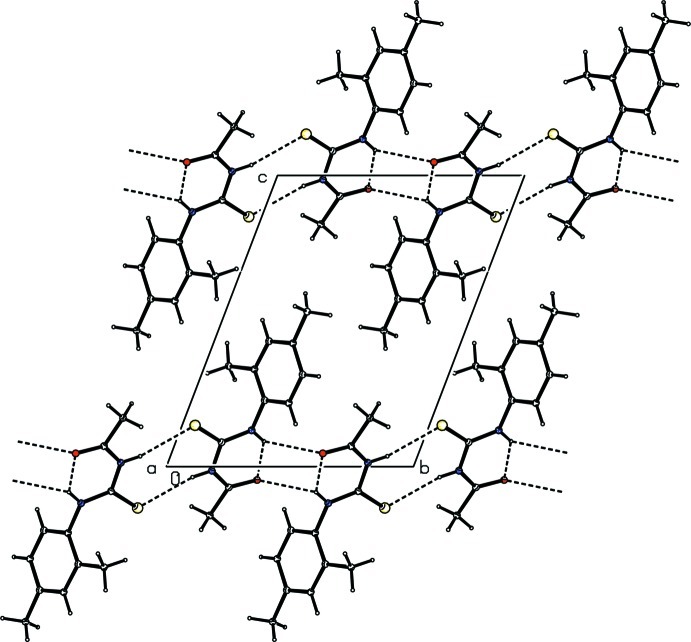
Molecular packing of the title compound with hydrogen bonding shown as dashed lines.

## supplementary crystallographic information

### Comment

Thiourea and its derivatives are known to exhibit a variety of biological activities. As part of our studies on the substituent effects on the structures and other aspects of *N*-(aryl)-amides (Gowda *et al.*, 2001; Kumar *et al.*, 2012: Shahwar *et al.*, 2012); *N*-(aryl)-methanesulfonamides (Gowda *et al.*, 2007) and *N*-chloroarylsulfonamides (Gowda & Ramachandra, 1989; Shetty & Gowda, 2004), in the present work, the crystal structure of 3-acetyl-1-(2,4-dimethylphenyl)thiourea has been determined (Fig. 1).

The conformation of the two N—H bonds are *anti* to each other. The conformations of the amide C═S and the C═O are also *anti* to each other and both the bonds are *anti* to the adjacent N—H bonds, similar to the *anti* conformation observed in 3-acetyl-1-(2,3-dimethylphenyl)thiourea (I)(Kumar *et al.*, 2012). The adjacent N—H bond is *anti* to the *ortho*-methyl group, similar to the *anti* conformation observed with respect to the *ortho*- and *meta*-methyl groups in the benzene ring of (I).

The side chain is oriented itself with respect to the phenyl ring with the C2—C1—N1—C7 and C6—C1—N1—C7 torsion angles of -76.98 (21)° and 105.91 (19)°, compared to the corresponding values of 83.59 (47)° and -99.89 (44)° in (I). The dihedral angle between the phenyl ring and the side chain is 77.5 (1)°, compared to the value of 81.3 (1)° in (I).

The NH hydrogen atom adjacent to the phenyl ring and the amide oxygen are involved in bifurcated hydrogen bonding, exhibiting the simultaneous intra- and inter-molecular hydrogen bonding. In the crystal structure, series of N—H···O and N—H···S intermolecular hydrogen bonds (Table 1) link the molecules into R_2_^2^(8) and R_2_^2^(12) networks (Fig.2).

### Experimental

3-Acetyl-1-(2,4-dimethylphenyl)thiourea was synthesized by adding a solution of acetyl chloride (0.10 mol) in acetone (30 ml) dropwise to a suspension of ammonium thiocyanate (0.10 mol) in acetone (30 ml). The reaction mixture was refluxed for 30 min. After cooling to room temperature, a solution of 2,4-dimethylaniline (0.10 mol) in acetone (10 ml) was added and refluxed for 3 h. The reaction mixture was poured into acidified cold water. The precipitated title compound was recrystallized to constant melting point from acetonitrile. The purity of the compound was checked and characterized.

Prism like colourless single crystals used in X-ray diffraction studies were grown in acetonitrile solution by slow evaporation of the solvent at room temperature.

### Refinement

H atoms bonded to C were positioned with idealized geometry using a riding model with aromatic C—H = 0.93 Å and methyl C—H = 0.96 Å. The coordinates of the amino H atoms were refined with the N—H distances restrained to 0.86 (2) Å. All H atoms were refined with isotropic displacement parameters set at 1.2 *U*_eq_(C-aromatic, N) and 1.5 *U*_eq_ (C-methyl) of the parent atom.

### Crystal data

**Table:**

C_11_H_14_N_2_OS	*Z* = 2
*M**_r_* = 222.30	*F*(000) = 236
Triclinic, *P*1	*D*_x_ = 1.262 Mg m^−^^3^
Hall symbol: -P 1	Mo *K*α radiation, λ = 0.71073 Å
*a* = 5.0510 (7) Å	Cell parameters from 2430 reflections
*b* = 9.973 (1) Å	θ = 3.3–27.8°
*c* = 12.503 (2) Å	µ = 0.25 mm^−^^1^
α = 69.15 (1)°	*T* = 293 K
β = 89.43 (1)°	Prism, colourless
γ = 84.07 (1)°	0.44 × 0.44 × 0.20 mm
*V* = 585.18 (14) Å^3^	

### Data collection

**Table:**

Oxford Diffraction Xcalibur diffractometer with a Sapphire CCD detector	2393 independent reflections
Radiation source: fine-focus sealed tube	2094 reflections with *I* > 2σ(*I*)
Graphite monochromator	*R*_int_ = 0.008
Rotation method data acquisition using ω and phi scans.	θ_max_ = 26.4°, θ_min_ = 3.3°
Absorption correction: multi-scan (*CrysAlis RED*; Oxford Diffraction, 2009)	*h* = −6→6
*T*_min_ = 0.897, *T*_max_ = 0.951	*k* = −12→12
3701 measured reflections	*l* = −14→15

### Refinement

**Table:**

Refinement on *F*^2^	Primary atom site location: structure-invariant direct methods
Least-squares matrix: full	Secondary atom site location: difference Fourier map
*R*[*F*^2^ > 2σ(*F*^2^)] = 0.037	Hydrogen site location: inferred from neighbouring sites
*wR*(*F*^2^) = 0.097	H atoms treated by a mixture of independent and constrained refinement
*S* = 1.07	*w* = 1/[σ^2^(*F*_o_^2^) + (0.0402*P*)^2^ + 0.2499*P*] where *P* = (*F*_o_^2^ + 2*F*_c_^2^)/3
2393 reflections	(Δ/σ)_max_ < 0.001
145 parameters	Δρ_max_ = 0.20 e Å^−^^3^
2 restraints	Δρ_min_ = −0.19 e Å^−^^3^

### Special details

**Table:**

Experimental. Abosrption correction: CrysAlis RED (Oxford Diffraction, 2009) Empirical absorption correction using spherical harmonics, implemented in SCALE3 ABSPACK scaling algorithm.
Geometry. All e.s.d.'s (except the e.s.d. in the dihedral angle between two l.s. planes) are estimated using the full covariance matrix. The cell e.s.d.'s are taken into account individually in the estimation of e.s.d.'s in distances, angles and torsion angles; correlations between e.s.d.'s in cell parameters are only used when they are defined by crystal symmetry. An approximate (isotropic) treatment of cell e.s.d.'s is used for estimating e.s.d.'s involving l.s. planes.
Refinement. Refinement of *F*^2^ against ALL reflections. The weighted *R*-factor *wR* and goodness of fit *S* are based on *F*^2^, conventional *R*-factors *R* are based on *F*, with *F* set to zero for negative *F*^2^. The threshold expression of *F*^2^ > σ(*F*^2^) is used only for calculating *R*-factors(gt) *etc*. and is not relevant to the choice of reflections for refinement. *R*-factors based on *F*^2^ are statistically about twice as large as those based on *F*, and *R*- factors based on ALL data will be even larger.

### Fractional atomic coordinates and isotropic or equivalent isotropic displacement parameters (Å^2^)

**Table:**

	*x*	*y*	*z*	*U*_iso_*/*U*_eq_	
S1	1.11565 (9)	0.05240 (5)	0.14186 (4)	0.04794 (16)	
O1	0.4331 (3)	0.38737 (13)	−0.04561 (11)	0.0535 (4)	
N1	0.7972 (3)	0.28872 (14)	0.12507 (11)	0.0372 (3)	
H1N	0.676 (3)	0.3551 (19)	0.0874 (15)	0.045*	
N2	0.7355 (3)	0.19052 (15)	−0.01371 (11)	0.0368 (3)	
H2N	0.785 (4)	0.1221 (18)	−0.0360 (16)	0.044*	
C1	0.9086 (3)	0.29552 (17)	0.22808 (13)	0.0371 (4)	
C2	0.8314 (3)	0.20597 (19)	0.33431 (14)	0.0418 (4)	
C3	0.9404 (4)	0.2218 (2)	0.43090 (16)	0.0528 (5)	
H3	0.8925	0.1627	0.5030	0.063*	
C4	1.1165 (4)	0.3217 (2)	0.42369 (18)	0.0578 (5)	
C5	1.1864 (5)	0.4092 (2)	0.3166 (2)	0.0620 (6)	
H5	1.3043	0.4772	0.3101	0.074*	
C6	1.0835 (4)	0.39718 (19)	0.21850 (17)	0.0494 (4)	
H6	1.1315	0.4570	0.1467	0.059*	
C7	0.8707 (3)	0.18561 (16)	0.08396 (13)	0.0337 (3)	
C8	0.5208 (3)	0.28452 (17)	−0.07079 (13)	0.0383 (4)	
C9	0.4051 (4)	0.2509 (2)	−0.16736 (16)	0.0524 (5)	
H9A	0.3200	0.1639	−0.1369	0.079*	
H9B	0.5448	0.2382	−0.2166	0.079*	
H9C	0.2767	0.3291	−0.2103	0.079*	
C10	0.6410 (4)	0.0967 (2)	0.34550 (17)	0.0568 (5)	
H10A	0.7238	0.0220	0.3208	0.085*	
H10B	0.4841	0.1426	0.2989	0.085*	
H10C	0.5928	0.0553	0.4240	0.085*	
C11	1.2348 (6)	0.3306 (3)	0.5323 (2)	0.0884 (9)	
H11A	1.4040	0.2730	0.5507	0.133*	
H11B	1.1165	0.2952	0.5943	0.133*	
H11C	1.2583	0.4291	0.5203	0.133*	

### Atomic displacement parameters (Å^2^)

**Table:**

	*U*^11^	*U*^22^	*U*^33^	*U*^12^	*U*^13^	*U*^23^
S1	0.0495 (3)	0.0487 (3)	0.0531 (3)	0.01791 (19)	−0.0211 (2)	−0.0330 (2)
O1	0.0681 (8)	0.0424 (7)	0.0520 (7)	0.0189 (6)	−0.0248 (6)	−0.0253 (6)
N1	0.0456 (8)	0.0348 (7)	0.0329 (7)	0.0092 (6)	−0.0116 (6)	−0.0174 (6)
N2	0.0423 (7)	0.0373 (7)	0.0362 (7)	0.0055 (6)	−0.0082 (6)	−0.0222 (6)
C1	0.0430 (9)	0.0359 (8)	0.0359 (8)	0.0103 (7)	−0.0104 (7)	−0.0205 (7)
C2	0.0411 (9)	0.0480 (9)	0.0375 (9)	0.0084 (7)	−0.0061 (7)	−0.0203 (7)
C3	0.0572 (11)	0.0653 (12)	0.0359 (9)	0.0138 (9)	−0.0089 (8)	−0.0233 (9)
C4	0.0681 (12)	0.0603 (12)	0.0554 (12)	0.0191 (10)	−0.0265 (10)	−0.0398 (10)
C5	0.0752 (14)	0.0469 (11)	0.0726 (14)	−0.0021 (10)	−0.0257 (11)	−0.0323 (10)
C6	0.0624 (11)	0.0386 (9)	0.0501 (10)	−0.0018 (8)	−0.0110 (8)	−0.0199 (8)
C7	0.0362 (8)	0.0341 (8)	0.0334 (8)	−0.0004 (6)	−0.0032 (6)	−0.0162 (6)
C8	0.0452 (9)	0.0359 (8)	0.0337 (8)	0.0014 (7)	−0.0081 (7)	−0.0137 (7)
C9	0.0600 (11)	0.0536 (11)	0.0476 (10)	0.0087 (9)	−0.0224 (9)	−0.0262 (9)
C10	0.0529 (11)	0.0671 (13)	0.0461 (10)	−0.0090 (10)	0.0012 (8)	−0.0144 (9)
C11	0.114 (2)	0.0906 (18)	0.0769 (16)	0.0238 (16)	−0.0485 (15)	−0.0576 (15)

### Geometric parameters (Å, º)

**Table:**

S1—C7	1.6774 (16)	C4—C11	1.524 (3)
O1—C8	1.217 (2)	C5—C6	1.385 (3)
N1—C7	1.3239 (19)	C5—H5	0.9300
N1—C1	1.4369 (19)	C6—H6	0.9300
N1—H1N	0.856 (14)	C8—C9	1.502 (2)
N2—C8	1.376 (2)	C9—H9A	0.9600
N2—C7	1.3883 (19)	C9—H9B	0.9600
N2—H2N	0.840 (14)	C9—H9C	0.9600
C1—C6	1.385 (2)	C10—H10A	0.9600
C1—C2	1.390 (2)	C10—H10B	0.9600
C2—C3	1.398 (2)	C10—H10C	0.9600
C2—C10	1.496 (3)	C11—H11A	0.9600
C3—C4	1.382 (3)	C11—H11B	0.9600
C3—H3	0.9300	C11—H11C	0.9600
C4—C5	1.379 (3)		
			
C7—N1—C1	123.94 (13)	N1—C7—N2	117.04 (13)
C7—N1—H1N	117.4 (13)	N1—C7—S1	124.11 (12)
C1—N1—H1N	118.6 (13)	N2—C7—S1	118.86 (11)
C8—N2—C7	128.32 (13)	O1—C8—N2	122.67 (14)
C8—N2—H2N	117.3 (13)	O1—C8—C9	122.70 (15)
C7—N2—H2N	114.1 (13)	N2—C8—C9	114.64 (14)
C6—C1—C2	121.37 (15)	C8—C9—H9A	109.5
C6—C1—N1	118.48 (15)	C8—C9—H9B	109.5
C2—C1—N1	120.09 (15)	H9A—C9—H9B	109.5
C1—C2—C3	117.08 (17)	C8—C9—H9C	109.5
C1—C2—C10	121.75 (15)	H9A—C9—H9C	109.5
C3—C2—C10	121.17 (17)	H9B—C9—H9C	109.5
C4—C3—C2	122.68 (19)	C2—C10—H10A	109.5
C4—C3—H3	118.7	C2—C10—H10B	109.5
C2—C3—H3	118.7	H10A—C10—H10B	109.5
C5—C4—C3	118.36 (17)	C2—C10—H10C	109.5
C5—C4—C11	121.5 (2)	H10A—C10—H10C	109.5
C3—C4—C11	120.1 (2)	H10B—C10—H10C	109.5
C4—C5—C6	120.95 (19)	C4—C11—H11A	109.5
C4—C5—H5	119.5	C4—C11—H11B	109.5
C6—C5—H5	119.5	H11A—C11—H11B	109.5
C5—C6—C1	119.55 (19)	C4—C11—H11C	109.5
C5—C6—H6	120.2	H11A—C11—H11C	109.5
C1—C6—H6	120.2	H11B—C11—H11C	109.5
			
C7—N1—C1—C6	105.91 (19)	C11—C4—C5—C6	178.1 (2)
C7—N1—C1—C2	−77.0 (2)	C4—C5—C6—C1	−0.2 (3)
C6—C1—C2—C3	−0.8 (2)	C2—C1—C6—C5	0.7 (3)
N1—C1—C2—C3	−177.78 (14)	N1—C1—C6—C5	177.79 (16)
C6—C1—C2—C10	179.50 (17)	C1—N1—C7—N2	177.11 (15)
N1—C1—C2—C10	2.5 (2)	C1—N1—C7—S1	−3.4 (2)
C1—C2—C3—C4	0.3 (3)	C8—N2—C7—N1	−4.4 (3)
C10—C2—C3—C4	−179.90 (17)	C8—N2—C7—S1	176.05 (14)
C2—C3—C4—C5	0.1 (3)	C7—N2—C8—O1	6.3 (3)
C2—C3—C4—C11	−178.16 (18)	C7—N2—C8—C9	−174.26 (16)
C3—C4—C5—C6	−0.2 (3)		

### Hydrogen-bond geometry (Å, º)

**Table:**

*D*—H···*A*	*D*—H	H···*A*	*D*···*A*	*D*—H···*A*
N1—H1*N*···O1	0.86 (1)	1.99 (2)	2.6673 (18)	135 (2)
N1—H1*N*···O1^i^	0.86 (2)	2.44 (2)	3.121 (2)	137 (2)
N2—H2*N*···S1^ii^	0.84 (1)	2.55 (2)	3.3711 (14)	168 (2)

Symmetry codes: (i) −*x*+1, −*y*+1, −*z*; (ii) −*x*+2, −*y*, −*z*.
